# Eluviation and Leaching of Elements from Broken Fly-Ash-Based Porous Geopolymer

**DOI:** 10.3390/ma14226884

**Published:** 2021-11-15

**Authors:** Peng Shi, Yuan Zhang, Qingfu Sun, Xupeng Ta

**Affiliations:** 1 School of Mines, China University of Mining & Technology, Xuzhou 221116, China; sqf19851625664@163.com (Q.S.); TB19020025B0@cumt.edu.cn (X.T.); 2 Department of New Energy Science & Engineering, China University of Mining & Technology, Xuzhou 221116, China

**Keywords:** fly-ash-based porous geopolymer, dynamic eluviation, static leaching

## Abstract

The fly ash from powerplants used for coal mine end backfilling can effectively reduce the impact of ground fly ash accumulation on the environment. However, due to the long-term action of the overlying strata and groundwater, when the backfilling body is broken, heavy metals will also be leached, thus having an impact on the groundwater. Therefore, in this paper, the eluviation and leaching of elements from a broken fly-ash-based porous geopolymer is studied. The fly-ash-based geopolymer material was prepared to perform a dynamic eluviation and static leaching test, and it was found that the amount of Cu and Zn in the leachate was less abundant, whereas Pb was more abundant, but far less than the limit of the Class III groundwater quality standard. An acidic environment and a smaller solid–liquid ratio can promote the leaching of Cu and Zn, while the leaching of Pb is basically unaffected by the pH value. Moreover, the amount of Cu, Zn, and Pb in the lixivium increased with the increase in leaching time, and the amount of Cu and Zn in the lixivium was still low after 150 h of leaching, whereas the amount of Pb was high, approaching the limit value of the Class III groundwater quality standard, showing a tendency to increase after 100 h of leaching. A leaching orthogonal experiment was designed, and the results showed that the main order of each factor affecting the leaching of heavy metals from the fly-ash-based geopolymer was grain size > pH > solid–liquid ratio; thus, the leaching of heavy metals from fly-ash-based geopolymer can be controlled, which is significant with respect to the extensive use of fly-ash materials underground.

## 1. Introduction

Driven by the concept of a green environment, the coal–electricity integrated industry model has become a major development strategy for coal and electricity companies [1,2,3]. An important part of this industrial model is the backfilling of powerplant fly ash underground [4,5,6], which is mainly used for the reinforcement of surrounding rock mass [7,8,9], face end plugging [10], and spontaneous ignition of remnant coal within the gob [11,12]. Meanwhile, in order to prevent air leakage of the coal face and fires in the gob [13,14,15,16,17], the coal face ends need to be backfilled. Therefore, face end backfilling represents one of the main sources of mine fly-ash destruction.

**Figure 1:**
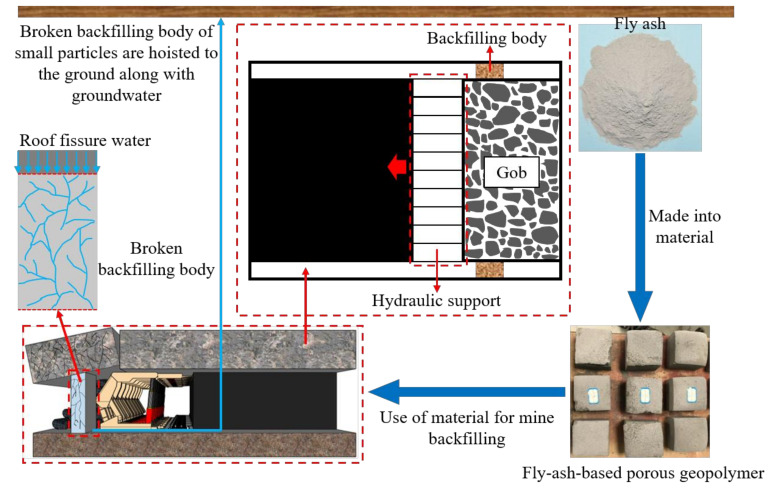
Face end backfilling in the coal–electricity integrated industry model.

However, with the continuous advancement of the coal face, its roof water-conducting fissures continue to expand, leading to the backfilling body being leached by roof fissure water in contact with it. At this time, under the pressure of the overlying strata, the backfilling body is prone to produce larger crushing, and the broken backfilling body of small particles will be lifted to the ground, together with the recycling of groundwater, thus becoming subject to a longer immersion in groundwater, as shown in Figure 1. The above process leads to the leaching of heavy metals within the backfilling body, which increases the difficulty of groundwater recycling treatment. Therefore, the study of eluviation and leaching of elements from broken fly-ash-based porous geopolymer is a great significance for the large-scale use of fly ash underground.

Regarding eluviation and leaching of elements from fly-ash-based concrete, many scholars have conducted studies. Among them, Madzivire [18] found that the migration of pollutants between fly ash and AMD (mine drainage) depended on the pH of the mine water by using fly ash as a backfilling material and placing it in underground leaching. Qi [19] confirmed that fly ash as a grouting material can cause fluorine contamination in groundwater through a fly ash F-72h leaching test. Zheng [20] found that mine backfilling can cause heavy-metal contamination in groundwater by conducting simulation experiments. Wang [21] analyzed the leaching characteristics of trace elements in coal gangue–fly ash sintered bricks through a leaching test and found that the maximum leaching amounts of Co, Ni, and As in the leaching solution were 1.8 times, 2.8 times, and 4.6 times the corresponding limits of the Class III groundwater environmental quality standards, respectively, which caused greater potential hazards to the environment. Yu [22] analyzed the effect of different ratios on the leaching of heavy metals from concrete by adjusting the fly-ash cement mortar and cement-cured fly ash mixing ratio, leaching agent pH, curing time, and specimen preparation method, finding that the leaching of heavy metals increased with the mass of fly ash incorporated and the water–cement ratio. Xu [23] showed that the leaching of As, Cd, Cu, Pb, and Cr was strongly inhibited by a fly-ash-based curing agent compounded with slaked lime, but it had no effect on the migration of Ni and Zn.

The leaching pattern of heavy metals in fly-ash-based concrete can be obtained by adjusting the fly-ash admixture, leachate pH, leaching time, and other conditions. However, little research has been reported on the leaching pattern of heavy metals from broken fly-ash-based porous geopolymer with different grain sizes. In this study, we conducted dynamic eluviation tests with different grain sizes and eluviation times, as well as static leaching tests with different grain sizes, pH values, solid–liquid ratios, and leaching times. The paper is organized as follows: the engineering background is introduced in Section 2, the materials and methods are outlined in Section 3, and the results and discussion are presented in Section 4. Lastly, several important conclusions are drawn in Section 5.

## 2. Engineering Background

### 2.1. Overview of Coal Face

**Figure 2:**
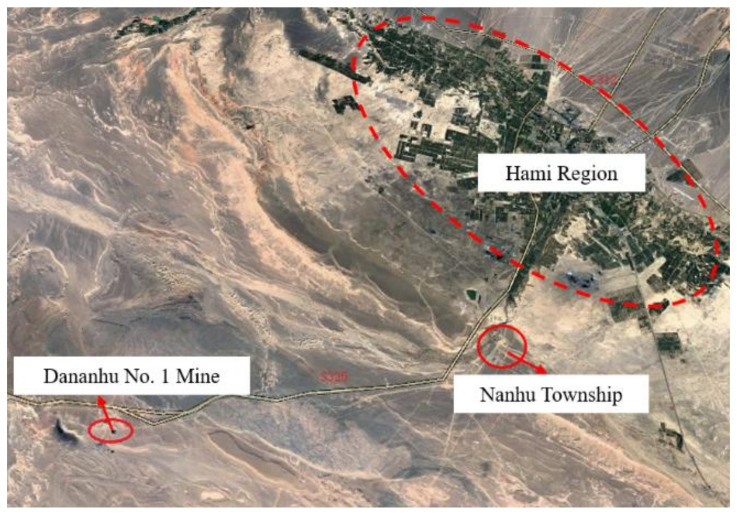
Traffic location of Dananhu No. 1 Coal Mine.

**Figure 3:**
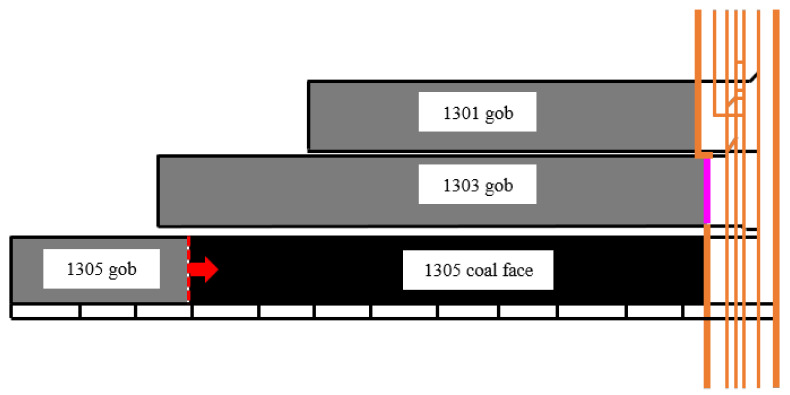
Location of 1305 coal face.

The Dananhu No. 1 Coal Mine is located in the eastern part of the Dananhu Coalfield, about 50 km from the Nanhu Township. The Lanzhou–Xinjiang Railway and the Gansu–Xinjiang Highway pass along the east side of the mine from the northwest to the southeast, with the traffic location shown in Figure 2. The 1305 coal face is located in the west limb of No. 1 district. The fully mechanized full-seam top coal caving mining method was employed. The heights of mining and top coal caving were 2.8–3.0 m and 6.7–7.2 m, respectively. The layout of the 1305 coal face was 238 m wide by 3100 m long. The location of the 1305 coal face is shown in Figure 3.

### 2.2. Overview of Face End Backfilling

**Figure 4:**
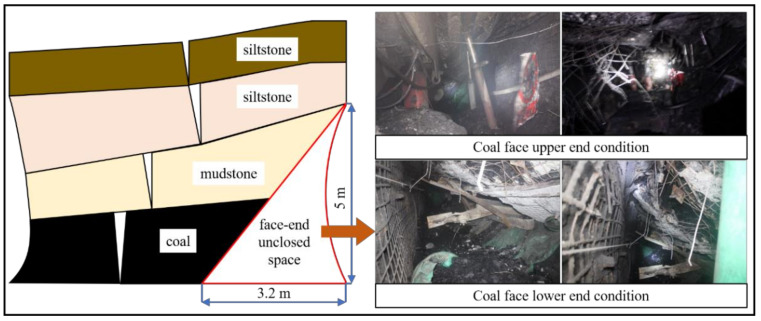
**The** 1305 coal face end diagram.

Since the coal face is advancing, the overlying roof strata of the 1305 gob is collapsing, thereby forming a triangular section at the face end (as shown in Figure 4), which results in oxygen entering the gob with the wind flow, causing spontaneous ignition of the remnant coal within it. The ventilation volume was tested at the face end of the 1305 coal face using a CFJD-25 type air flow meter, which found that the air leakage volume in this area was large and could reach 14.25 m^3^/min. Therefore, it is urgent to seal the 1305 coal face end.

### 2.3. Environment of Backfilling Body Eluviation

The main water-filled sources of the 1305 coal face consist of sandstone water in the roof of the No. 3 coal seam, abandoned mine water around the 1305 coal face, and abandoned mine water from the 1303 coal face.

The sandstone aquifers can be divided as follows: an aquifer of coarse sand and sandy conglomerates from the Jurassicsystem Middle Xishangyao Formation Middle Section, and a sandstone aquifer from the Jurassicsystem Middle Xishangyao Formation Upper Tip Bottom Boundary. The former is the direct water-filled aquifer of No. 3 coal, which is a weak water-bearing layer with developed rock fissures. The latter is the main water-filled water source of the 1305 working face.

Located in the north side of 1305 coal face, the water accumulation area and the volume of the 1303 gob are 43,335 m^2^ and 93,600 m^3^, respectively.

There are five gobs distributed around the 1305 coal face, whose area can reach 2.66 km^2^, and the static storage capacity of the groundwater in them is 5004 × 104 m^3^.

The vertical rupture fissure is more developed in the range of 30 m on the roof of the coal seam. The coal face end backfilling body is located on the side of the overburden fissure from the fissure field, and it produces a large fissure due to the influence of roof subsidence on the process of coal extraction, which results in the coal face end backfilling body being significantly affected by the water in the vertical rupture fissure, making it subject to a strong groundwater eluviation effect.

## 3. Materials and Methods

### 3.1. Materials

Fly ash, 425 ordinary portland cement, and additives (sodium hydroxide, calcium stearate, anhydrous calcium sulfate, and calcium oxide) were selected as raw materials in this test. Fly ash was from Shenhua Guoneng Hami Dananhu Plant, Hami, China, sodium hydroxide and anhydrous calcium sulfate contributed to form the early strength of the fly-ash materials, calcium stearate was mainly used as a foam stabilizer, and calcium oxide was used as a quick setting agent for the cement.

**Figure 5:**
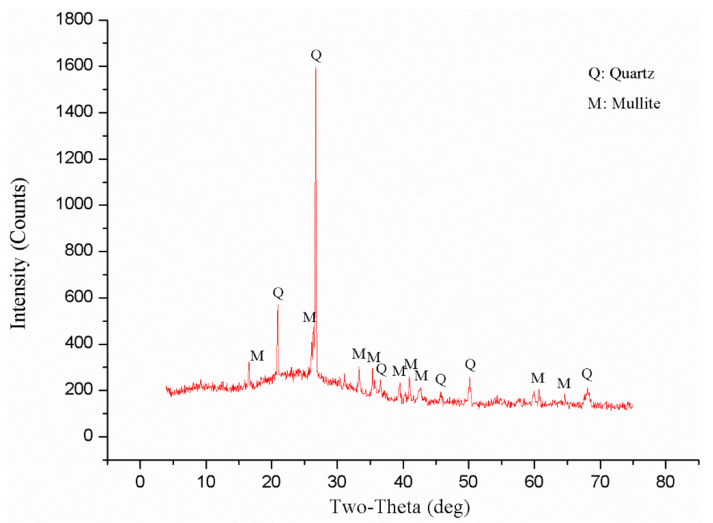
Fly-ash XRD ray spectrum.

The fly-ash mineral composition and its content were calculated and analyzed by XRD and XRF (The analyzer is X-ray diffractometer D8 ADVANCE from BRUKER, Germany), with the results shown in Figure 5 and Table 1. As can be seen, the main components in the fly ash were SiO_2_, Al_2_O_3_, and Fe_2_O_3_, which together accounted for 88.3% of the total content, followed by a certain amount of CaO, MgO, Na_2_O, and K_2_O. In addition, the content of CaO in the original ash was less than 10%, which was, thus, characterized as Class III low-calcium fly ash (Class F).

### 3.2. Fly-Ash-Based Porous Geopolymer Preparation

**Figure 6:**
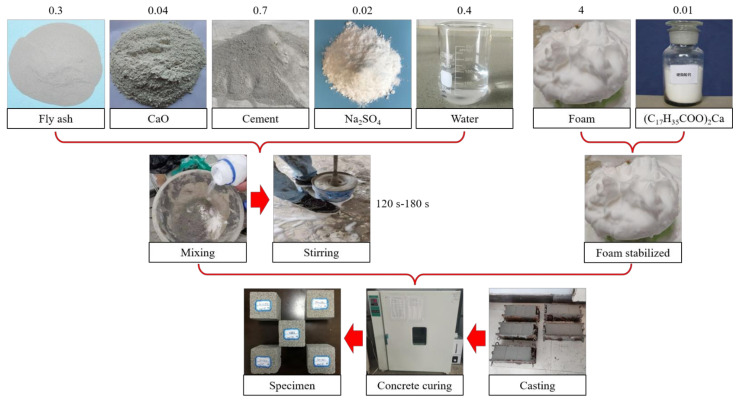
Preparation of fly-ash-based porous geopolymers and the proportioning of raw materials (note: the percentages were obtained from the ratios of masses of corresponding materials to the total mass of cement and fly ash).

The fly-ash-based porous geopolymer was prepared as shown in Figure 6. Firstly, the foam was formulated according to a 1:40 ratio of foaming agent to water, using the physical foaming method. Then, the fly ash, cement, additives, and water were combined according to the ratio scheme and stirred well for 120–180 s. Next, the foam was added before mixing well again. Finally, the mixed slurry was poured in a 100 mm × 100 mm × 100 mm triplex test mold, and the sample was demolded after 48 h. Meanwhile, the demolded specimens were placed in a maintenance box (It was produced by Zhejiang Lichen Instrument Technology Company Limited, Zhejiang Province, China) for 28 days at a constant temperature of 20 ± 2 ℃ and humidity > 95%.

To characterize the leaching regularity of heavy metals under different broken forms of the backfilling body, the specimens were broken after concrete curing, followed by sieving them with sieves of different mesh numbers: 20–35 mesh, 35–60 mesh, and > 60 mesh.

### 3.3. Testing Apparatus

**Figure 7:**
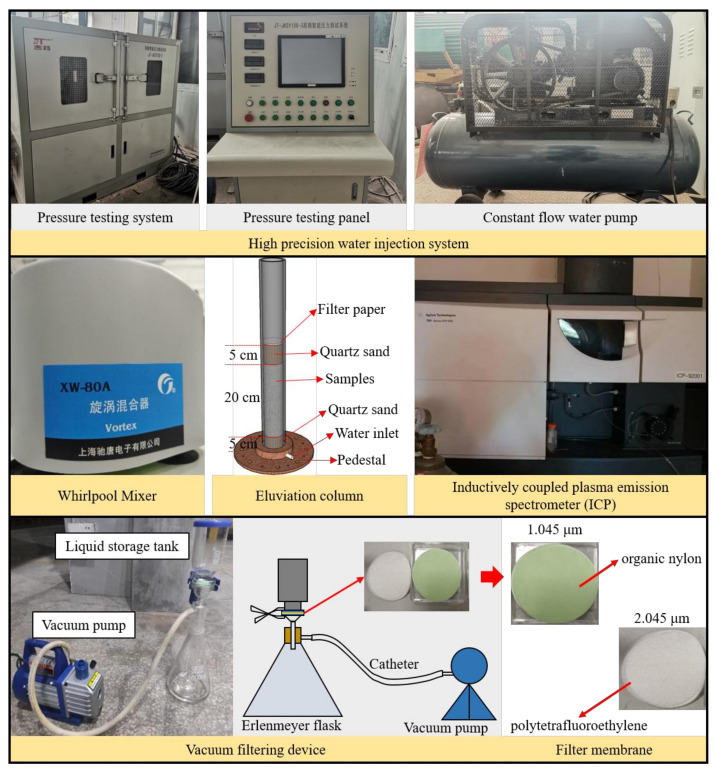
Testing apparatus.

The testing apparatus required for this study is shown in Figure 7. Dynamic eluviation tests were conducted using a high-precision water injection system and eluviation tube, static leaching tests were conducted using a vortex mixer, the leachate or lixivium was filtrated using a filtration device, and the heavy metals in the leachate or lixivium were detected using an inductively coupled plasma emission spectrometer (ICP) produced by Wuxi Cai Da Materials Technology Company Limited in Wuxi, Jiangsu Province, China.

### 3.4. Experimental Content and Procedures

According to the preliminary experimental data, the leachate contained high contents of Pb, Cu, Zn, and Ni, but the Ni content was relatively low and less toxic; therefore, Pb, Zn, and Cu were the objects of study in this paper. The eluviation and leaching process of heavy metals in the underground backfilling body is very complicated owing to the constantly changing environment; thus, it was simplified in this paper.

(1) Simplification of eluviation process

The leachate rate was set to 30 mL/min in this paper, and the grain size of the fly-ash-based geopolymer used in the same group of tests was the same for convenience of analysis.

(2) Simplification of leaching process

The oscillation frequency was set to 2800 times/min, and the vibration time was set to 10 min in this paper.

(3) Simplification of mine water

The factors influencing the leaching regularity of heavy metals in the fly-ash-based geopolymer were identified by controlling the test variables. Thus, deionized water was selected for eluviation and leaching tests in this paper.

#### 3.4.1. Dynamic Eluviation Test

The dynamic eluviation test scheme was designed as shown in Table 2. Before the test, the eluviation tube was filled with quartz sand at a height of 5 cm, and then filled with geopolymer particles at a height of 20 cm, followed by quartz sand at a height of 5 cm and a filter paper on top of it.

During the test, deionized water was flowed from the bottom of the eluviation tube, and the leachate was collected into the reservoir; then, the leachate was filtered into the Erlenmeyer flask (It was produced by Jiangsu Shunhe Teaching Instruments Company Limited in Taizhou City, Jiangsu Province, China). Finally, the Pb, Cu, and Zn contents in the leachate were detected.

#### 3.4.2. Static Leaching Test

**Figure 8:**
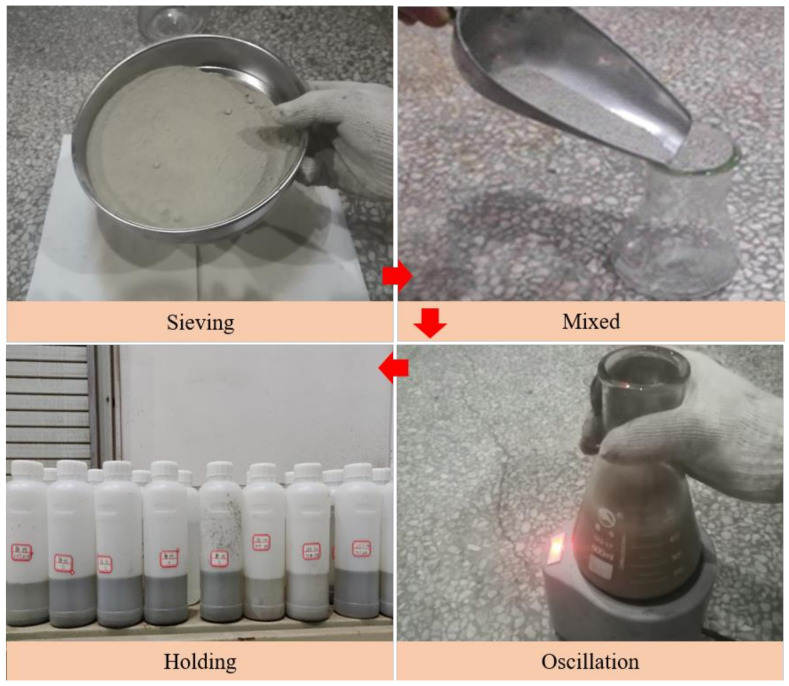
Static leaching test procedure.

The static leaching test scheme was designed in this paper as shown in Table 3. Firstly, 500 mL of lixivium was added to the Erlenmeyer flask, which contained deionized water, acidic buffer (pH = 2, 4), and alkaline buffer (pH = 10, 12). Then, 50 g of fly-ash-based geopolymer particles were added, and the Erlenmeyer flask was shaken on a mixer. Subsequently, it was left for a certain time at a constant temperature according to the test scheme, and the contents of Pb, Cu, and Zn in the lixivium were finally detected. The test procedure is shown in Figure 8.

#### 3.4.3. Leaching Orthogonal Experiment

The leaching orthogonal test of fly-ash-based geopolymer particles was designed according to a L_9_(3^4^) orthogonal table in this paper. The mixing, oscillating, holding, filtering, and heavy metal detection were completed sequentially according to the steps of the leaching test above, and the orthogonal test is shown in Table 4.

## 4. Results and Discussion

### 4.1. Analysis of Dynamic Eluviation Test Results

**Figure 9:**
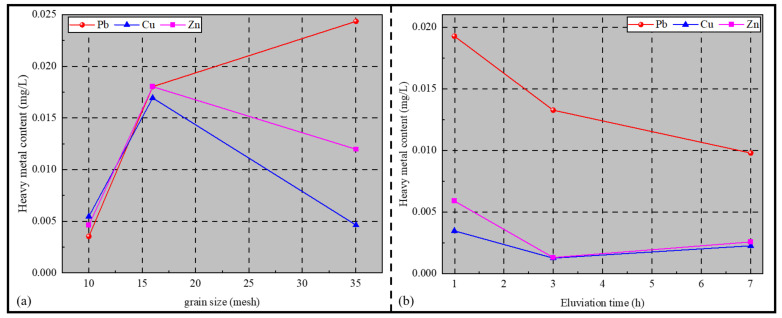
Dynamic eluviation test results: (**a**) Heavy metal content versus grain size curve, (**b**) Heavy metal content versus eluviation time curve.

The results of the dynamic eluviation test are shown in Figure 9a,b. Firstly, the amount of Cu and Zn in the leachate was low, while that of Zn was high. Secondly, with the increase in eluviation time, the amount of Cu and Zn in the leachate decreased first and then increased slightly. Thirdly, with the increase in grain size, the amount of Cu and Zn in the leachate first increased and then decreased. Fourthly, with the increase in eluviation time, the amount of Pb in the leachate gradually decreased. Lastly, with the increase in grain size, the amount of Pb in the leachate gradually decreased.

The three heavy metals had different leaching regularities, mainly related to the form of their presence within the fly-ash-based geopolymer structure. Halim [24] and Jingchuan [25] et al. suggested that Pb ions are homogeneously dispersed in the cement matrix, and most of the Pb is emitted via the surface of water by forming compounds with other elements, such that it can be easily leached out. Gineys et al. [26] suggested that Cu^2+^ and Zn^2+^ in the cementitious material combine with C–S–H via chemical bonding, thus changing the C–S–H crystal structure. This results in the presence of Zn and Cu mainly in the form of solid melts within the structure of fly-ash-based geopolymer, which exhibit chemical bonding during the formation of the geopolymer, leading to Zn and Cu not being easily leached out.

A smaller grain size of the geopolymer particles leads to a larger total specific surface area, resulting in a higher contact probability between Pb and the leachate, which makes it easier to be leached out. However, with the eluviation process, Pb was continuously leached out by the leachate, reducing the amount of Pb adsorbed on the surface of the geopolymer, which resulted in a decreasing concentration of Pb in the leachate. However, the reduction rate of Pb concentration in the leachate was slowed at the later stage since the leachate became dilute. Meanwhile, CO_2_ was dissolved in the leachate with the eluviation process, making it weakly acidic, resulting in the destruction of the chemical bond formed by Cu and Zn in the leachate, which caused a slight increase in the amount of Cu and Zn in the leachate at the later stage of eluviation.

### 4.2. Analysis of Static Leaching Test Results

**Figure 10:**
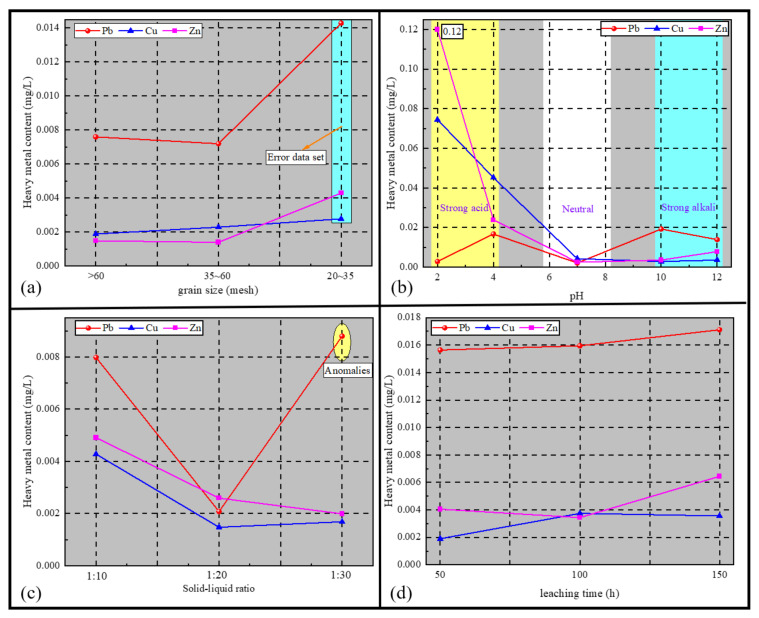
Static leaching test results: (**a**) Heavy metal content versus grain size curve, (**b**) Heavy metal content versus leachate pH curve, (**c**) Heavy metal content versus Solid-liquid ratio curve, (**d**) Heavy metal content versus leaching time curve.

The results of the static leaching test are shown in Figure 10a–d. From the figure, it can be seen that the Cu and Zu contents in the leaching solution only fluctuated slightly (±0.002 mg/L) with the increase in grain size, indicating that the effect of grain size on the leaching of Cu and Zu was relatively small. The amount of Pb in the lixivium reached about 50% of the limit value, and, with the increase in grain size, the amount of Pb in the lixivium decreased slightly and then increased significantly, which was different from the conclusion obtained from the eluviation test, probably because the fly-ash-based geopolymer contained a certain amount of clay minerals. Thus, under the effect of oscillation, the smaller-sized geopolymer particles were easily bonded together, resulting in the inhibition of Pb leaching, whereas the degree of bonding between the larger-grain-size geopolymer particles was smaller, which had less of an effect on the leaching of Pb.

The chemical bonds formed by Cu and Zn in the fly-ash-based geopolymer were more easily broken in an acidic environment, leading to an increase in their leaching. In particular for Cu, its leaching increased by several orders of magnitude in the strongly acidic environment compared to the weakly acidic environment. In the neutral and alkaline environment, the concentration of Cu and Zn in the lixivium was smaller. However, in the more alkaline environment (pH >10), a large amount of OH^−^ in the lixivium formed carboxylate complexes such as Zn(OH)^3−^ and Zn(OH)2−4 with Zn^2+^ in the materials, resulting in a slight increase in the Zn concentration in the lixivium compared with the weak alkaline environment.

The leaching of Pb was basically not affected by pH and did not show a significant geometric increase or decrease under strong acidic or strong alkaline environments, but its leaching was slightly larger under weakly acidic (weakly alkaline) conditions, reaching 0.02 mg/L. Since mine water is mostly weakly acidic (weakly alkaline), the leaching of Pb under the action of groundwater is of concern.

With the decrease in the solid–liquid ratio, the volume of the lixivium increased, which decreased the concentration of Cu and Zn, but the decreasing trend gradually became slower, indicating that the increase in lixivium promoted the leaching of Cu and Zn. With the decrease in solid–liquid ratio, the concentration of Pb in the lixivium decreased and then increased, probably because the leaching rate of Pb was not greatly affected when the solid–liquid ratio was reduced from 1:10 to 1:20; however, the volume of the lixivium increased nearly twofold, resulting in a decrease in Pb concentration in the lixivium. When the solid–liquid ratio was reduced to 1:30, the leachate was in full contact with the ground polymer particles, which led to a significant increase in the leaching rate of Pb; thus, the concentration of Pb increased.

The concentrations of Cu, Zn, and Pb in the lixivium increased with the increase in leaching time, and the contents of Cu and Zn in the lixivium were still low after 150 h of leaching, which were far lower than the Class III groundwater quality standard. The amount of Cu basically reached a stable state after 100 h of leaching, whereas Zn and Pb still had a tendency to increase after 100 h of leaching, and the amount of Pb was higher and close to the limit value of the Class III groundwater quality standard.

### 4.3. Analysis of Orthogonal Experimental Results

**Figure 11:**
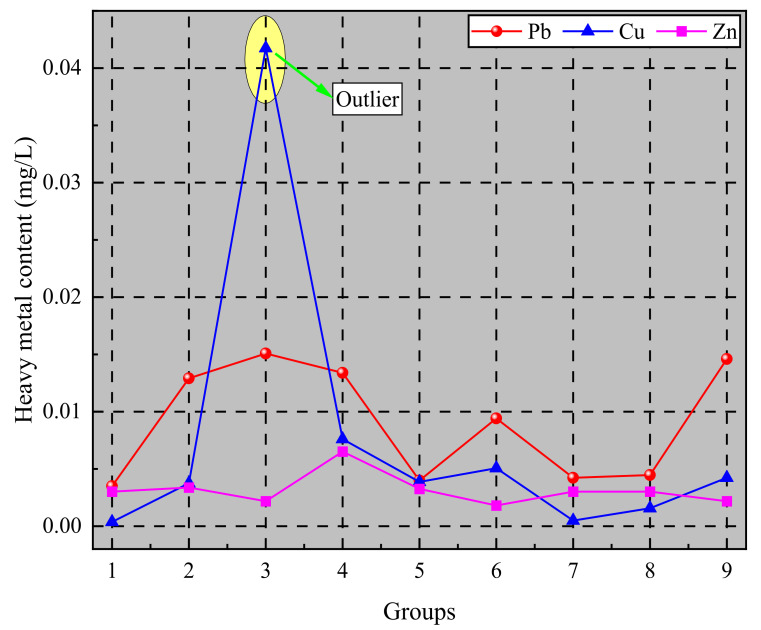
Leaching orthogonal experimental results.

The leaching patterns of Pb, Cu, and Zn from fly-ash-based geopolymer tested in each group were obtained by orthogonal experiments, as shown in Figure 11. 

As can be seen from Figure 10, the amount of Pb, Cu, and Zn in the lixivium was small, but the leaching of Cu increased abnormally in the third group of tests, at which time the amount of Pb in the lixivium reached its maximum, showing that pH has a large effect on the leaching of heavy metals in fly-ash-based geopolymer.

To identify the order in which factors affect the leaching regularity of heavy metals in fly-ash-based geopolymer, the leaching of Pb, Cu, and Zn was analyzed by range analysis, as shown in Table 5.

As can be seen from Table 5, the order of factors affecting the leaching of heavy metals from fly-ash-based geopolymer was grain size > pH > solid–liquid ratio. The total specific surface of fly-ash-based geopolymer increased during the process of being broken under pressure, causing more heavy metals adsorbed on the surface of the fly ash geopolymer to be leached. At the same time, under the acidic environment, the lixivium had a certain corrosive effect on the chemical bonds formed by the fly-ash-based geopolymer, thus leading to the leaching of a large amount of heavy metals. Moreover, the solid–liquid ratio had less of an influence on the leaching of heavy metals from fly-ash-based geopolymer.

## 5. Conclusions

The following remarks can be commonly applied to the leaching of heavy metals from broken fly-ash-based geopolymers: heavy metals leached from broken fly-ash-based geopolymers; the leachability of heavy metals in this study depended largely on the geopolymer grain size, the pH of the leachate (lixivium), and the test method; the leaching rate of heavy metals increased for smaller grain sizes and lower pH, but the effect of grain size and leachate (lixivium) pH on the leaching behavior of heavy metals was dependent on the specific heavy metals; the content of heavy metals in the leachate (lixivium) was below the III groundwater quality standard. Some other observations and remarks of this study are listed below.

(1) The amount of Cu and Zn in the leachate was low, and the concentration of Cu and Zn in the leachate decreased and then increased slightly with the increase in eluviation time, but their concentration increased and than decreased with the increase in grain size.
(2) The concentration of Pb in the leachate was greater, and it decreased gradually with the increase in leaching time. However, it gradually decreased with the increase in grain size.
(3) Under acidic environments, the leaching of Cu and Zn became easier, especially Cu, which increased by several orders of magnitude in strongly acidic environments compared to weakly acidic environments. Under neutral and alkaline environments, the amount of Cu and Zn in the lixivium was lower. However, the amount of Zn in the leachate increased slightly in the strongly alkaline environment (pH > 10) compared to the weakly alkaline environment.
(4) The leaching of Pb was basically not affected by pH and did not show an obvious geometric increase or decrease in either a strongly acidic or a strongly alkaline environment, but its leaching amount was slightly larger under weakly acidic (weakly alkaline) conditions, which could reach 0.02 mg/L.
(5) The reduction in solid–liquid ratio promoted the leaching of heavy metals from fly-ash-based geopolymer.
(6) The order of the factors affecting the leaching of heavy metals from fly-ash-based geopolymer was grain size > pH > solid–liquid ratio.

## Figures and Tables

**Table 1:** Main chemical composition of fly ash.

Component	SiO_2_	Al_2_O_3_	Fe_2_O_3_	CaO	K_2_O	Na_2_O	MgO	TiO	Others
Ratio	58.3%	24.6%	5.4%	4.6%	2.0%	2.0%	1.5%	1.1%	0.5%

**Table 2:** Dynamic eluviation test scheme.

Groups	Grain Size (Mesh)	Eluviation Time (h)
1	20–35/35–60/>60	3
2	20–35	1/3/7

**Table 3:** Static leaching test scheme.

Groups	Solid-to-Liquid Ratio	Grain Size (Mesh)	Lixivium pH	Leaching Time (Days)
1	1:10	20–35/35–60/>60	7	3
2	1:10	20–35	2/4/7/10/12	3
3	1:10/1:20/1:30	20–35	7	3
4	1:10	20–35	7	2/4/6

**Table 4:** Leaching orthogonal experiment.

Groups	pH	Grain Size (Mesh)	Solid-to-Liquid Ratio
1	7.00	35–60	1:30
2	7.00	>60	1:20
3	4.00	>60	1:30
4	10.00	>60	1:10
5	4.00	35–60	1:10
6	7.00	20–35	1:10
7	10.00	35–60	1:20
8	10.00	20–35	1:30
9	4.00	20–35	1:20

**Table 5:** Range analysis of heavy metal leaching from fly-ash-based geopolymer.

Name of Heavy Metal	Analysis Parameters	Influencing Factors		
		pH (A)	Solid-to-Liquid Ratio (B)	Mesh Number (C)
Pb	K_1_	0.0336	0.0267	0.0117
	K_2_	0.0255	0.0318	0.0285
	K_3_	0.0222	0.0228	0.0411
	${\bar{K}}_{1}$	0.0112	0.0089	0.0039
	${\bar{K}}_{2}$	0.0085	0.0106	0.0095
	${\bar{K}}_{3}$	0.0074	0.0076	0.0137
	Optimum level	A_1_	B_2_	C_3_
	*R_j_*	0.0038	0.003	0.0098
	Primary and secondary order	C > A > B		
Cu	K_1_	0.0498	0.0165	0.0048
	K_2_	0.0090	0.0081	0.0108
	K_3_	0.0096	0.0435	0.0528
	${\bar{K}}_{1}$	0.0166	0.0055	0.0016
	${\bar{K}}_{2}$	0.0030	0.0027	0.0036
	${\bar{K}}_{3}$	0.0032	0.0145	0.0176
	Optimum level	A_1_	B_3_	C_3_
	*R_j_*	0.0133	0.0118	0.0160
	Primary and secondary order	C > A > B		
Zn	K_1_	0.0075	0.0114	0.0090
	K_2_	0.0078	0.0084	0.0069
	K_3_	0.0123	0.0081	0.0120
	${\bar{K}}_{1}$	0.0025	0.0038	0.0030
	${\bar{K}}_{2}$	0.0026	0.0028	0.0023
	${\bar{K}}_{3}$	0.0041	0.0027	0.0040
	Optimum level	A_3_	B_1_	C_3_
	*R_j_*	0.0016	0.0011	0.0017
	Primary and secondary order	C > A > B		

## Data Availability

The data used to support the findings of this study are available from the corresponding author upon request.
